# Morphine plasmatic concentration in a pregnant mare and its foal after long term epidural administration

**DOI:** 10.1186/s12917-020-2242-9

**Published:** 2020-01-20

**Authors:** Alessandro Mirra, Jasmin Birras, Sabina Diez Bernal, Claudia Spadavecchia

**Affiliations:** 1 Department of Clinical Veterinary Medicine, Anaesthesiology and Pain Therapy Section, Vetsuisse Faculty, University of Bern, Länggassstrasse 124, 3012 Bern, Switzerland; 2 Swiss Institute for Equine Medicine (ISME), Department of Clinical Veterinary Medicine, Vetsuisse Faculty, University of Bern, and Agroscope, Bern, Switzerland

**Keywords:** Horse, Foal, Pregnancy, Epidural, Morphine, Morphine-3-glucuronide, Morphine-6-glucuronide

## Abstract

**Background:**

Epidural administration of morphine has been shown to be an effective analgesic strategy in horses; however, the possible occurrence of side effects limits its usage. In order to decrease their frequency, it is important to target the minimal effective plasma concentration and avoid overdosing. As to date species-specific pharmacokinetics data are not available for epidural morphine, the dosing regimen is usually established on the basis of clinical reports and personal experience. In certain physiological conditions, like gestation, the outcome of an empirical dosing scheme can be unpredictable. The aim of this case report is to describe the pharmacological profile of morphine and its metabolites after prolonged epidural administration in a pregnant mare and her foal.

**Case presentation:**

A 20 years old pregnant mare was presented to our hospital because of severe lameness, 2 months before delivery. Following an ineffective systemic pain treatment, an epidural catheter was inserted and morphine administered (initial dose 0.1 mg/kg every 8 h). Due to its efficacy in controlling pain, it was continued until end of gestation. Plasmatic concentration of morphine and its metabolites were assessed in the mare 6 weeks after starting the treatment, and in both the mare and foal during the first days after delivery. Plasmatic values similar to those previously reported in the literature following morphine short term administration through various routes and not accompanied by side effects were found in the mare, except during an excitatory period. Moreover, no evidence of dangerous drug accumulation or significant milk passage was noticed in the foal. Mild reduction of feces production with no signs of colic and two self-limiting episodes of excitement occurred during treatment in the mare. No side effects occurred during gestation and first phases of life in the foal.

**Conclusion:**

Prolonged epidural administration of morphine in a pregnant mare allowed good pain control in absence of clinically relevant side effects, in both the mare and her foal. Sudden increase in morphine plasmatic concentration can occur and side effects appear; careful treatment to the lowest effective dose and continuous monitoring of the clinical condition of the treated horse should be performed.

## Background

Epidural injection of morphine has been used to treat various painful conditions in horses [1–3]. Despite good clinical outcomes after both single and repeated injections, the risk of side effects, especially on the gastrointestinal system, limit its usage [4]. However, it has recently been shown that a single dose of epidural morphine (up to 0.1 mg/kg) does not delay gastro-intestinal transit time [5]. Moreover, repeated epidural administration of morphine (0.1 mg/kg every 8 h for 4 days) did not provoke any neurological or behavioural abnormalities [3]. Finally, no changes in rectal temperature, heart rate, respiratory rate, and gastrointestinal motility were observed during prolonged epidural administration over a period of 14 days (0.2 mg/kg of morphine every 12 h) [6].

Morphine is primarily metabolised by the liver. The only metabolites identified in equine blood are morphine-3- glucuronide (M3G) and morphine-6-glucuronide (M6G) [7]. While M6G has been shown to have analgesic activity [8], animal studies suggest that M3G has no analgesic effect [9] and may antagonise the analgesic effect of M6G and morphine [10]. Moreover, it has been associated with neuro-excitatory behaviour in rats [11].

When drug administration is performed in pregnant mares, it is important to consider that drug absorbed into the systemic circulation can reach the foetus. Valverde et al. (1990) [1] reported a successful repeated epidural injection over three days in a pregnant mare following digital amputation; unfortunately, no specific information about effects on the foetus as well as no pharmacological profile was described.

The aim of this case report is to describe the pharmacological profile of morphine and its metabolites after repeated epidural injections in a pregnant mare, 6 weeks after start of the treatment. Moreover, plasmatic concentrations of morphine and its metabolites in the foetus after birth are reported.

## Case presentation

A 550 Kg, 20 years old pregnant mare was presented at the equine teaching hospital due to severe hindlimbs lameness. A heart rate of 56 beats per minute, a respiratory rate of 16 breaths per minute and clear signs of pain were present at the clinical examination. A radiological study revealed a high-grade, chronic tendinopathy of the suspensory branches on the right and left side. Since foal delivery was expected within 2 months, the horse was hospitalised for pain and supportive therapy. During the hospitalization, pain could not be easily controlled, despite the adoption of a multimodal systemic analgesic plan. A pain score [12] was used in order to monitor the analgesic efficacy of the attempted treatments. Due to the inefficacy of a combination of methadone, non-steroidal anti-inflammatory agents and gabapentin in controlling pain over the first week, an epidural catheter placement was planned, in order to provide repeated morphine administrations into the epidural space. The sacro-coccigeal area was clipped and aseptically prepared, and a Tuohy needle was inserted targeting the sacro-coccigeal space, with the needle opening pointing cranially. The hanging drop technique with sterile saline was used to confirm the needle tip location into the epidural space. An epidural catheter was advanced through the Tuohy needle, for a length previously calculated in order to reach the L4-L5 vertebra. A bacterial filter was applied to the injection port and the catheter was secured to the skin with adhesive material and covered with adhesive dressing. The systemic analgesic treatment was stopped. An initial epidural dose of 0.1 mg/kg of morphine (Morphin HCl Sintetica, Sintetica S.A., Switzerland) every 8 h was started, with immediate improvement of the clinical condition. After each drug injection, the epidural catheter was always flushed with a volume of 6 ml of sterile saline. An abdominal ultrasound examination was repeatedly performed in order to check the clinical condition of the foal, with no abnormalities detected until delivery. On day 16 (from now on, days are reported as “days after starting the epidural analgesic treatment”), a strong excitatory phase with increased locomotor activity, dysphoria and photophobia occurred and lasted for few hours. Even if it could not be correlated to any particular injection or time interval after injection, a morphine overdose was suspected. Its administration was stopped and epidural methadone (0.1 mg/kg) (Methadone Sintetica, Sintetica S.A., Switzerland) was given instead. Two days later, due to stable clinical conditions, methadone was stopped and morphine restarted. During the excitatory period, the horse was moved to an isolated and quiet stable and the windows were covered with black light-reflecting material. On day 21, due to a weaning of the analgesic effect of morphine within one hour before administration, shorter intervals (6 h) were adopted and ketamine (Ketasol, Graeub AG, Switzerland) was added (0.1 mg/kg every 6 h). On day 42, a second but milder excitatory episode occurred; also in this case, it could not be correlated to any particular injection or time interval after injection. Morphine was interrupted for 24 h and it was substituted by epidural methadone (0.1 mg/kg). The day after, due to stable clinical conditions, methadone was stopped and morphine restarted at half of the dosage. On day 49, the foal was delivered and morphine dose was further reduced to 0.025 mg/kg. On day 53, epidural administration was stopped and morphine (0.025 mg/kg) and ketamine (0.5 mg/kg) were injected intramuscularly, every 8 h. On day 56 the epidural catheter was removed. A bacterial examination of the catheter tip was performed and no contamination was found. Finally, on day 57, the mare and the foal were discharged from the hospital in stable clinical conditions and the analgesic therapy of the mare was continued at home by the private veterinarian. During the whole hospitalization period, reduction in faeces production occurred but no signs of colic were noticed.

### Blood sampling

After obtaining owner consent, the plasmatic levels of morphine ant its metabolites (M3G and M6G) were evaluated to titrate the analgesic treatment over time. Moreover, blood samples were taken from the foal (in concomitance with already needed blood withdrawal for routine tests) in order to rule out morphine overdosing. On day 36, a sequential blood sampling (SBS) from the mare was performed, at 30 min before and 1, 3 and 5 h after morphine administration. Another sample was taken on day 42, one hour and a half after morphine injection, during the episode of excitation and increased locomotor activity. Further blood samples were taken on day 49, 50 and 51 (day of delivery and 1 and 2 days post-delivery). For each blood collection, 10 ml of blood were withdrawn and put aside, 10 ml were then collected in an EDTA syringe and the previous blood administered back to the horse. The blood was immediately centrifuged (3000 rotation per minute for 10 min at 20 °C) and the plasma successively stored at − 80° in special cryotube (CryoPure Tube, Sarstedt, Germany). Withdrawal of blood from the foal was performed on day 0 (after delivery, before first meal), on day 1 and on day 2. In this case, 1.5 ml of blood was taken and transferred in an EDTA tube; then, the same centrifugation and storages process as for the mare was applied. Measurement of morphine, M3G and M6G plasmatic concentration were performed using liquid chromatography-tandem mass spectrometry. Results are presented in Tables 1 and 2.

**Table 1:** Plasmatic concentration of morphine and its metabolites in the mare (ng/ml)

Days	Morphine	M3G	M6G
36	5.88	213.87	7.59
36	19.89	307.85	12.96
36	8.25	278.57	10.36
36	6.16	241.21	8.48
42	63.18	149.26	9.45
49	1.66	93.01	3.08
50	4.82	125.20	5.79
51	1.66	57.82	2.23

**Table 2:** Plasmatic concentration of morphine and its metabolites in the foal (ng/ml)

Days (from birth)	Morphine	M3G	M6G
0	2.40	6.71	< 0.14
1	1.48	10.13	< 0.14
2	< 0.03	3.11	< 0.14

## Discussion and conclusion

Although the usefulness of epidural analgesia in horses is well documented in the literature, only scarce information about plasma concentrations of morphine following epidural administration is available.

So far, no pharmacokinetic studies about morphine and its metabolites after repeated epidural administration have been reported in horses. Natalini (2006) [13] investigated the plasmatic concentration of morphine after a single epidural injection of 0.1 mg/kg in horses and found maximum plasmatic concentration of 90 ng/ml at 120 min post-injection; no clinical variables were reported.

In another study, following intravenous (IV) and intramuscular (IM) administration of 0.05 or 0.1 mg/kg morphine in horses, values higher than 8 ng/ml were found and they were not correlated with any respiratory or behavioural modifications, except mild reduction of gut sounds (with no reduction in faeces production) [4]. However, in the same study, an increase in heart rate and blood pressure developed for approximately two minutes after IV injection, associated with morphine plasmatic level > 150 ng/ml.

In another study in horses, behavioural responses, such as fasciculations, sweating and increase in locomotor activity were noticed for about 45 min after morphine IV injection at dosages of 0.2 to 0.5 mg/kg [7].

To the authors’ knowledge, no available data on minimum effective plasmatic concentrations of morphine and its metabolites in equine species are known. In the present study, values between 6.4 and 21.9 ng/ml for morphine, 8.3 and 14.2 ng/ml for M6G and 235.6 and 339.2 ng/ml for M3G were found during the SBS. As successful analgesia was achieved in absence of clinically relevant side effects, plasmatic concentrations in the reported ranges could be considered as effective. Moreover, they did not exceed the level previously reported to induce tachycardia and increase in blood pressure in horses [7].

In the present case, when the SBC was performed, peak plasmatic concentration of morphine and its metabolites were found 1-h post injection and trough 5.5 h post injection (30 min before the next injection).

A strong agonism of M6G to mu-receptors have been reported [14–17]. Around 85% of the analgesic effect of morphine is supposed to derive from the M6G [18]. In contrast, M3G has up to 200 times lower mu-receptor binding compared with morphine [19] and it seems to lack analgesic activity, with some studies suggesting an antagonist [10, 16, 20] or just a weak agonist activity [21].

It is interesting to notice that when the blood sample was taken during the excitatory period, morphine concentration was 69.6 ng/ml, around three times higher than the peak plasmatic concentration found during the SBS, while M6G and M3G were consistent with the previously measured concentrations (10.4 ng/ml for M6G and 164.4 ng/ml for M3G). From these results it could be hypothesized that the high concentration of morphine, rather than its metabolites, could be the cause of the excitatory side effects, but the reason why there was an increase in morphine and not of its metabolites is not clear. Full blood work was performed the same day, but no pathological findings, especially in the liver function tests, were found.

Up to date, there were no reports describing plasmatic concentrations of morphine in neonatal foals born from mares receiving long-term epidural morphine. Moreover it is not known whether there might be a correlation between maternal morphine plasmatic concentration and the occurrence of undesired side effects in the new born foal. At the concentrations found in the present case, no side effects (respiratory depression, reduced appetite, dysphoria, among others) were seen, and no problems were encountered until discharge from the hospital. It is interesting to notice that plasmatic concentrations of morphine before the first meal were slightly higher in the foal compared to the mare, whereas plasmatic concentration of its metabolites were substantially higher in the mare compared to the foal. The immature liver function could have led to accumulation of the parent drug and reduction of its metabolization.

If the presence of morphine in the blood during the last phase of gestation can lead to pain pathway abnormalities/addiction in the future is not known for horses. In humans, neonates born from opioid dependent women are more likely to have low weight at birth, to be admitted to the neonatal intensive care unit and to require prolonged treatment for neonatal abstinence syndrome [22, 23]. The neonatal abstinence syndrome is a drug withdrawal syndrome after birth that affects 45–94% of infants born from opioid dependent mothers, resulting in significant neonatal morbidity [24–26]. Sometimes it can also appear after few days from birth [27]. Even though morphine has a low lipid solubility compared to other opioids, its poor protein binding favour its passage through the placenta. In the present case, no abnormalities were noticed during the hospitalization period and no abnormalities were reported from the owner up to 3 months after birth. The difference in placental structure between humans and horses, as well as the different maturity at birth could play an important role in drug transfer and effects on the foetus.

The possible passage from the mare to the foal during lactation should also be considered. In a case series of women undergoing surgery during the lactation period (at least 1 month after giving birth) receiving IV or IM morphine, only mild and occasionally observed respiratory depression or drowsiness in the child occurred [28]. In another clinical study in women receiving epidural morphine for delivery, measurable quantities in the milk were found only in three samples within the following 36 h [29]. In the present case no analysis of the milk was performed. However, plasmatic concentration of morphine and its metabolites in the foal decreased from day 0 (before first meal) to day 2, reflecting a possible null or minimal passage of the drug from the milk to the neonate.

A last point to consider is that differences in plasmatic volume during pregnancy could have led to different plasma concentrations compared to non-pregnant horses. Recently, a review about pharmacokinetic and pharmacodynamics of morphine in pregnant women has been published. Compared to non-pregnant women, morphine in pregnant women has an unchanged volume of distribution, a clearance > 70% and a decreased half-life [30]. In another study, the area under the curve for unconjugated morphine within two days after morphine epidural injection in pregnant women was similar to those reported for epidural administration in the non-pregnant ones [29]. To the authors knowledge, the influence of gestation on the morphine pharmacokinetics and pharmacodynamics in pregnant horses is not known.

This case report presents plasmatic concentrations of morphine and its metabolite after a long-term epidural treatment in a pregnant mare. Results show that plasmatic level reached over prolonged epidural administration do not exceed those previously reported to be effective and at the same time safe for the animal. Sudden increase in morphine plasmatic concentration in the mare can occur and side effect appear; careful treatment to the lowest effective dose and continuous monitoring of the clinical condition of the treated horse should be performed. No clinically relevant effects on the foetus were noticed during the whole gestation period; moreover, the plasmatic concentrations found after birth did not affect the essential steps during the first phases of life.

## Data Availability

The datasets used and/or analysed during the current study available from the corresponding author on reasonable request.

## Abbreviations

M3G: Morphine-3- glucuronide
M6G: Morphine-6-glucuronide
SBS: Sequential blood sampling

## References

[1] Valverde A, Little CB, Dyson DH, Motter CH (1990). Use of epidural morphine to relieve pain in a horse. Can Vet J.

[2] Goodrich LR, Nixon AJ, Fubini SL, Ducharme NG, Fortier LA, Warnick LD, Ludders JW (2002). Epidural morphine and detomidine decreases postoperative hindlimb lameness in horses after bilateral stifle arthroscopy. Vet Surg.

[3] Rodrigo-Mocholi D, Steblaj B, Vlaminck L, Gasthuys F, Schauvliege S (2016). Continuous caudal epidural analgesia for perioperative pain control after bilateral mastectomy in a mare. Vet Rec Case Rep.

[4] Figueiredo JP, Muir WW, Sams R (2012). Cardiorespiratory, gastrointestinal, and analgesic effects of morphine sulfate in conscious healthy horses. Am J Vet Res.

[5] Martin-Flores M, Campoy L, Kinsley MA, Mohammed HO, Gleed RD, Cheetham J (2014). Analgesic and gastrointestinal effects of epidural morphine in horses after laparoscopic cryptorchidectomy under general anesthesia. Vet Anaesth Analg.

[6] Sysel AM, Pleasant RS, Jacobson JD, Moll HD, Warnick LD, Sponenberg DP, Eyre P (1997). Systemic and local effects associated with long-term epidural catheterization and morphine-detomidine administration in horses. Vet Surg.

[7] Knych HK, Steffey EP, McKemie DS (2014). Preliminary pharmacokinetics of morphine and its major metabolites following intravenous administration of four doses to horses. J Vet Pharmacol Ther.

[8] Osborne R, Thompson P, Joel S, Trew D, Patel N, Slevin M (1992). The analgesic activity of morphine-6-glucuronide. Br J clin Pharmac.

[9] Shimomura K, Kamata O, Ueki S, Ida S, Oguri K, Yoshimura H, Tsukamoto H (1971). Analgesic effect of morphine Glucuronides. Tohoku J Exp Med.

[10] Smith MT, Watt JA, Cramond T (1990). Morphine-3-glucuronide--a potent antagonist of morphine analgesia. Life Sci.

[11] Hemstapat K, Monteith GR, Smith D, Smith MH (2003). Morphine-3-Glucuronide’s Neuro-excitatory effects are mediated via indirect activation of N-methyl-d-aspartic acid receptors: mechanistic studies in embryonic cultured hippocampal Neurones. Anesth Analg.

[12] Gleerup KB, Forkman B, Lindegaard C, Andersen PH (2015). An equine pain face. Vet Anaesth Analg.

[13] Natalini CC (2006). Plasma and cerebrospinal fluid alfentanil, butorphanol, and morphine concentrations following caudal epidural administration in horses. Ciência Rural.

[14] Frölich N, Dees C, Paetz C, Ren X, Lohse MJ, Nikolaev VO, Zenk MH (2011). Distinct pharmacological properties of morphine metabolites at G(i)-protein and β-arrestin signaling pathways activated by the human μ-opioid receptor. Biochem Pharmacol.

[15] Lötsch J, Geisslinger G (2001). Morphine-6-glucuronide: an analgesic of the future?. Clin Pharmacokinet.

[16] Mercadante S (1999). The role of morphine glucuronides in cancer pain. Palliat Med.

[17] Pasternak GW, Bodnar RJ, Clark JA, Inturrisi CE (1987). Morphine-6-glucuronide, a potent mu agonist. Life Sci.

[18] Hand CW, Blunnie WP, Claffey LP, McShane AJ, McQuay HJ, Moore RA (1987). Potential analgesic contribution from morphine-6-glucuronide in CSF. Lancet.

[19] Mignat C, Wille U, Ziegler A (1995). Affinity profiles of morphine, codeine, dihydrocodeine and their glucuronides at opioid receptor subtypes. Life Sci.

[20] Gong QL, Hedner T, Hedner J, Björkman R, Nordberg G (1991). Antinociceptive and ventilatory effects of the morphine metabolites: morphine-6-glucuronide and morphine-3-glucuronide. Eur J Pharmacol.

[21] Ulens C, Baker L, Ratka A, Waumans D, Tytgat J (2001). Morphine-6beta-glucuronide and morphine-3-glucuronide, opioid receptor agonists with different potencies. Biochem Pharmacol.

[22] Hulse GK, Milne E, English DR, Holman CD (1997). The relationship between maternal use of heroin and methadone and infant birth weight. Addiction.

[23] Madden JD, Chappel JN, Zuspan F, Gumpel J, Mejia A, Davis R (1977). Observation and treatment of neonatal narcotic withdrawal. Am J Obstet Gynecol.

[24] Dryden C, Young D, Hepburn M, Mactier H (2009). Maternal methadone use in pregnancy: factors associated with the development of neonatal abstinence syndrome and implications for healthcare resources. BJOG.

[25] Burns L, Conroy E, Mattick RP (2010). Infant mortality among women on a methadone program during pregnancy. Drug Alcohol Rev.

[26] Bandstra ES, Morrow CE, Mansoor E, Accornero VH (2010). Prenatal drug exposure: infant and toddler outcomes. J Addict Dis.

[27] Hudak M. L., Tan R. C. (2012). Neonatal Drug Withdrawal. PEDIATRICS.

[28] Feilberg VL, Rosenborg D, Broen Christensen C, Mogensen JV (1989). Excretion of morphine in human breast milk. Acta Anaesthesiol Scand.

[29] Zakowski MI, Ramanathan S, Turndorf H (1993). A two-dose epidural morphine regimen in cesarean section patients: pharmacokinetic profile. Acta Anaesthesiol Scand.

[30] Ansari J, Carvalho B, Shafer SL, Flood P (2016). Pharmacokinetics and pharmacodynamics of drugs commonly used in pregnancy and parturition. Anesth Analg.

[31] Klimas R, Mikus G (2014). Morphine-6-glucuronide is responsible for the analgesic effect after morphine administration: a quantitative review of morphine, morphine-6-glucuronide, and morphine-3-glucuronide. Br J Anaesth.

